# Sensory Processing and Cognitive Resilience in Nursing Home Residents

**DOI:** 10.1111/ggi.70315

**Published:** 2026-01-05

**Authors:** Yi‐Ching Chu, Chao‐Chun Huang

**Affiliations:** ^1^ Department of Ophthalmology Far Eastern Memorial Hospital New Taipei City Taiwan; ^2^ Department of Physical Medicine and Rehabilitation Far Eastern Memorial Hospital New Taipei City Taiwan; ^3^ Department of Physical Therapy and Assistive Technology National Yang Ming Chiao Tung University Taipei Taiwan

To the Editor,

We read with great interest the article by Aydemir et al., which highlights the significant yet often overlooked relationship between sensory processing patterns and cognitive function in nursing home residents [1]. As clinicians, we typically focus on sensory *acuity*—correcting vision with glasses or hearing with aids. However, this study makes a crucial contribution by excluding participants with uncorrectable impairments, thereby isolating the role of central sensory *processing*. This methodological choice allows for a fascinating interpretation: the deficits observed likely reside not in the eyes or ears, but in the brain's ability to integrate and filter information.

The finding that visual over‐responsiveness is negatively associated with cognition (*β* = −0.321) is clinically striking [1]. In our practice, we frequently encounter residents who appear cognitively intact in quiet settings but become confused or withdrawn in visually chaotic environments. This hypersensitivity may reflect age‐related changes in cortical inhibitory control. Research in senescent models suggests that a degradation of GABAergic inhibition in the visual cortex compromises the brain's ability to filter out random neural activity or “noise” [2]. When the aging brain cannot efficiently suppress this irrelevant visual noise, it must allocate scarce cognitive resources to basic perceptual management, leaving little “bandwidth” for executive functions. Clinically, this suggests that patient complaints of “visual overwhelm” or glare sensitivity should be viewed as potential early markers of central cognitive fragility, even when standard Snellen visual acuity is normal.

Conversely, the positive association between proprioceptive seeking and cognitive status (*β* = 0.305) challenges the pathologization of certain motor behaviors [1]. Behaviors often labeled as “agitation” or “restlessness”—such as pacing or seeking deep pressure—might actually represent an adaptive strategy to compensate for age‐related declines in proprioceptive sensibility [3]. By actively seeking sensation, these residents may be upregulating somatosensory feedback loops to maintain body schema integrity and drive plastic‐adaptive processes. This supports the use of active sensorimotor engagement (e.g., resistance exercises, weighted blankets) rather than sedation for “restless” residents who may simply be trying to “find” their bodies in space.

Finally, the identification of interoception as the strongest predictor of cognition (*β* = 0.391) suggests that the ability to perceive internal states is fundamental to cognitive maintenance [1]. Preserved interoception relies on the functional integrity of the anterior insula, a hub for human awareness and emotional regulation [4]. Maintaining this awareness may offer a buffer against the behavioral dysregulation often seen in dementia.

We commend Aydemir et al. for providing evidence that supports a shift toward “Sensory‐Smart” care. Reducing environmental visual noise while amplifying meaningful proprioceptive opportunities could be a vital, non‐pharmacological strategy to sustain cognitive vitality in institutional settings.

## Funding

The authors have nothing to report.

## Ethics Statement

The authors have nothing to report.

## Consent

The authors have nothing to report.

## Conflicts of Interest

The authors declare no conflicts of interest.

## Data Availability

Data sharing not applicable to this article as no datasets were generated or analyzed during the current study.

## References

[1] F. R. Aydemir , A. E. Ezber , M. N. Ö. Değerli , and O. Altuntaş , “The Mind and the Senses: Associations of Sensory Processing and Interoception With Cognitive Function in Older Adults in Nursing Homes,” Geriatrics and Gerontology International (2025): 1–7. 10.1111/ggi.70277 41360081

[2] A. G. Leventhal , Y. Wang , M. Pu , Y. Zhou , and Y. Ma , “GABA and Its Agonists Improved Visual Cortical Function in Senescent Monkeys,” Science 300, no. 5620 (2003): 812–815. 12730605 10.1126/science.1082874

[3] D. J. Goble , J. P. Coxon , N. Wenderoth , A. Van Impe , and S. P. Swinnen , “Proprioceptive Sensibility in the Elderly: Degeneration, Functional Consequences and Plastic‐Adaptive Processes,” Neuroscience and Biobehavioral Reviews 33, no. 3 (2009): 271–278. 18793668 10.1016/j.neubiorev.2008.08.012

[4] A. D. Craig , “How Do You Feel–Now? The Anterior Insula and Human Awareness,” Nature Reviews. Neuroscience 10, no. 1 (2009): 59–70. 19096369 10.1038/nrn2555

